# Digital PCR assays for quantifying trichothecene-producing *Fusarium* species, including *Fusarium langsethiae*, *F. poae*, and *F. sporotrichioides*, in oats

**DOI:** 10.1007/s00216-025-05840-0

**Published:** 2025-03-21

**Authors:** Subramani Natarajan, Diana Bucur, Steven Kildea, Fiona Doohan

**Affiliations:** 1 School of Biology and Environmental Science, Earth Institute, University College Dublin, Belfield, Dublin, Ireland; 2 Department of Crop Science, Teagasc Crops Environment and Land Use Programme, Carlow, Ireland

**Keywords:** QPCR, Mycotoxins, Fusarium head blight, *Avena sativa*, Trichothecene

## Abstract

*Fusarium* fungi cause Fusarium head blight (FHB) in oats, reducing yield and contaminating grains with harmful trichothecene mycotoxins. FHB symptoms in oats are often not visually distinct, necessitating alternative detection methods. We developed digital PCR (dPCR) assays as the most accurate DNA-based method to detect trichothecene-producing *Fusarium* species commonly found in oats. Building on existing quantitative PCR (qPCR) assays, we developed dPCR assays targeting all trichothecene producers (the *Tri5* gene), or specific to *F. langsethiae* (*Fl*), *F. poae* (*Fp*), and *F. sporotrichioides* (*Fs*). All targeted single copy genes, except *F. poae* which targeted rDNA which is a variable and multi-copy target (and hence not as reliable as the other assays for quantification). Optimized dPCR assays showed excellent linearity (*R*^*2*^ = 0.99) and greater resilience than qPCR to varying oat DNA concentrations. Overall, when comparing assay sensitivity using both fungal and field oat DNA extracts, dPCR assays were superior to qPCR for *Tri5*, *Fl*, and *Fs*, but the converse was true for *Fp*. Performance comparisons using field samples showed moderate to perfect agreement between qPCR and dPCR for *Tri5* and *Fl* (*κ* = 0.5 and 0.86) and poor agreement for *Fp* (*κ* = 0.00). Strong correlations were observed between the methods for *Tri5*, *Fl*, and *Fp* (*r* = 0.88–0.97), but unlike dPCR, qPCR did not detect *Fs* in any of the field samples. We conclude that the dPCR assays for *Tri5*, *Fl*, and *Fs* offer a reliable method for quantification while that for *Fp* is reliable for fungal detection but less reliable for quantification of the pathogen in field samples.

**Supplementary Information:**

The online version contains supplementary material available at 10.1007/s00216-025-05840-0.

## Introduction

Oats (*Avena sativa*), a member of the *Poaceae* family, is an important cereal crop widely used as both a human food and livestock feed. Oats contain β-glucan which has been shown to regulate blood glucose levels and reduce cholesterol levels [1, 2]. In recent years, there has been an increase in the amount and diversity of oat-based human food products on the market, with demand driven by their high-nutritional value, including carbohydrates, fiber, vitamins, minerals, and antioxidants [3, 4].

In the field, diseases reduce the yield and quality of oat grain. Fusarium head blight (FHB) disease, caused by *Fusarium* fungi, poses a significant threat to oat cultivation. This disease can reduce yield and seed germination, contaminating grain with harmful fungal mycotoxins including the trichothecenes deoxynivalenol (DON), T-2, and HT-2. These mycotoxins cause acute health problems in humans and animals, including vomiting, weight loss, feed refusal, immunosuppression, and anorexia [5]. Several species of *Fusarium* can infect oats and contaminate them with mycotoxins. Studies have shown that *F. graminearum* and *F. culmorum* contaminate oats grain with DON, while T-2 and HT-2 toxins are produced by *F. langsethiae* and *F. sporotrichioides* [6–10]. Several studies have reported that *F. poae* also contaminates oats with T-2 and H-T2 [11–13]. However, while *F. poae* can produce other trichothecenes, it was found to lack the TRI16 ortholog, which encodes a key acyltransferase necessary for T-2 toxin production [14]. Furthermore, no evidence of T-2/HT-2 toxin production was observed in axenic cultures of *F. poae*. The European Commission has established legal regulations for *Fusarium* mycotoxins, setting maximum levels of 1750 μg/kg for deoxynivalenol (DON) and 1250 μg/kg for the sum of T-2 and HT-2 toxins in unprocessed oat grains with husks. Levels are lower in more processed cereals: 600 μg/kg of DON in milled cereal products and 100 μg/kg combined T2/HT-2 levels for oats placed on the market for the consumer [15, 16].

In oats, the visual symptoms of FHB disease can be easily mistaken for physiological changes, making this an unreliable method for identifying and quantifying FHB infection [17, 18]. *F. langsethiae* infection of oats is frequently asymptomatic, and apparently healthy grain can contain mycotoxins [19–21]. Traditional cultural and morphological approaches to diagnose *Fusarium* infection of grains are laborious and time-consuming and require expertise in fungal taxonomy. These approaches face challenges such as (i) overlapping morphological traits, complicating species differentiation; (ii) competitive growth in culture, leading to underestimation of slower-growing species; and (iii) inaccurate pathogen quantification on non-selective media [22–24]. Additionally, the accurate detection and quantification of specific *Fusarium* species pose significant challenges. For these reasons, molecular techniques such as PCR and quantitative PCR (qPCR) have been developed as cost-effective, sensitive, and rapid approaches for the accurate detection and quantification of toxigenic *Fusarium* species. Assays targeting the *Tri5* gene that encodes the trichodiene synthase enzyme have been used to detect trichothecene-producing *Fusarium* spp. [25–27]. Species-specific assays targeting nuclear ribosomal DNA (rDNA), which is present in multiple copies per genome, have been used to quantify *F. poae* [27, 28]. Species-specific assays targeting the single copy translation elongation factor 1-α (TEF1α) gene have been used to quantify other *Fusarium* species, including *F. graminearum*, *F. culmorum*, *F. poae*, *F. langsethiae*, *F. avenaeceum*, *F. tricinctum*, and *F. sporotrichioides* [29, 30].

In recent years, digital PCR (dPCR) technology has increasingly replaced qPCR for the quantification of nucleic acid targets, with the advantage that it directly measures copy numbers rather than extrapolating quantities from external standard curves, thus increasing the accuracy of measurement [31, 32]. dPCR target quantitation is absolute: the sample is divided into thousands of small individual reactions, and the copy numbers are determined through the application of Poisson distribution statistics to the positive and negative partitions measured by the fluorescence detector. This technique offers several advantages over qPCR, including direct quantification without a standard curve, increased sensitivity and precision, and less susceptibility to PCR inhibitors [33–35]. dPCR is being increasing used for phytopathogen diagnostics in agricultural crops and is seen as an effective and sensitive absolute quantification strategy: assays have been developed for some of the *Fusarium* species commonly found in cereals, including *F. graminearum*, *F. culmorum*, *F. sporotrichioides*, *F. poae*, and *F. avenaceum* [36, 37]. But there is no published assay for *F. langsethiae*, nor have any of the existing *Fusarium* dPCR assays been validated for use in oat grain.

The objective of this study was to develop dPCR assays and show their potential to detect and quantify trichothecene-producing *Fusaria* and specific *Fusarium* species in oat grain from field samples. To detect all trichothecene-producing species, we targeted the first gene in the trichothecene biosynthesis pathway, the *Tri5* gene (encoding trichodiene synthase); species-specific assays for *F. langsethiae*, *F. poae*, and *F. sporotrichioides* targeted TEF1α while that for *F. poae* targeted rDNA. All assays were based on primers and probes previously designed for qPCR [28–30]. Assays were optimized for annealing temperature and primer/probe concentration, and the potential of these assays to detect these targets in oat grain was demonstrated and compared with qPCR in terms of linearity, sensitivity, amplification efficiency in the presence of DNA extracts, and ability to detect and quantify fungi within field samples.

## Experimental procedures

### Origin and maintenance of fungal strains

This study used 18 *Fusarium* strains, including *Fusarium langsethiae* (3), *F poae* (4), *F. graminearum* (3), *F. sporotrichioides* (2), *F. avenaceum* (3), *F. culmorum* (3), and three strains of *Microdochium nivale* (Table S1). These strains were obtained from the UCD Molecular-Plant Microbe laboratory culture collection, and were originally obtained from Ireland, Hungary, Italy, Norway, and the UK, as detailed in Table S1. Fungal mycelial stocks were stored in 10% glycerol at −80 °C and, prior to use, they were sub-cultured on potato dextrose agar medium (DIFCO Laboratories, USA) and incubated at 20 °C for 7 days in the dark.

### Collection of field and glasshouse oat samples

Oat samples were collected from 20 Irish farmers field sites in 2022 for PCR analyses. Panicles were collected at harvest time (plant maturity). One bulk sample was collected per field comprising 50–80 oat panicles (depending on the field size) systematically sampled every 10 or 20 m along tramlines that were 24 m apart. Panicles were immediately placed in an ice box and transported back to the laboratory. Panicles were threshed and sieved to separate the grains. Grains were stored at −20 °C.

 Oats (cv. Husky) were grown under controlled glasshouse conditions at Rosemount Environmental Research Station, UCD, as previously described [38], to obtain disease-free DNA for spiking PCR assays. Briefly, the plants were cultivated in John Innes No. 2 compost (Westland Horticulture, UK), regularly watered, and a slow-release fertilizer (Osmocote Topdress FT 4-5M, ICL, UK) was applied 6 weeks after planting (2 g/l). Panicles were harvested upon maturity, and the seeds were threshed, cleaned, and stored at −20 °C.

### DNA extraction

For fungi, mycelium was scraped from PDA cultures and 100 mg was flash-frozen using liquid nitrogen and ground to a fine powder using a mortar and pestle. Fungal DNA was extracted using the plant DNA mini kit (Qiagen, USA) according to the manufacturer’s protocol. For oats, hulled grains obtained from glasshouse trials and from field samples were milled using a coffee grinder (BODUM® AG, Switzerland). DNA was extracted from 200 mg of finely ground oat flour using the Diatheva grain DNA extraction kit (Diatheva, Italy) as per the manufacturer’s protocol. The quality and concentration of DNA were determined spectrophotometrically using a Nanodrop (Bio drop, USA), and samples were stored at −20 °C.

### Primers and probes

A generic assay targeting trichothecene-producing *Fusarium* species (*Tri5* gene, referred to as the *Tri5* assay) and species-specific assays targeting *F. langsethiae*, *F. poae*, and *F. sporotrichioides* (hereafter referred to as the *Fl, Fp* and *Fs* assays, respectively) were used in this study. The primers and probes used were those previously developed for qPCR assays and are detailed in Table S2. Probes were labelled and quenched as follows: (i) TmTrip (for *Tri5*) was labelled at the 5′ end with the VIC dye and quenched at the 3′ end with the Minor Groove Binder (MGB) - non-fluorescent quencher (NFQ) (Applied Biosystems, USA); (ii) EF1_*FL* (*F. langsethiae*) and TMpoae (*F. poae*) were labelled at the 5′ end with the 6-carboxyfluorescein (FAM) dye and quenched with MGB-NFQ at the 3′ end (Applied Biosystems, USA); (iii) EF1_*FS* (*F. sporotrichioides*) was labelled at the 5′ end with the 6-carboxyfluorescein (FAM) dye and quenched with the Black Hole Quencher (BHQ) at the 3′ end (Metabion, Germany).

### Real-time qPCR conditions

The primer pairs and probe for each assay were first evaluated for reliability and amplification efficiency using real-time quantitative (qPCR), as described in [39]. Taqman assays were performed in a MicroAmp™ Fast Optical 96-Well Reaction Plate (Applied Biosystems, USA) sealed with adhesive covers on a Quant Studio 7 flex system (Applied Biosystems). Each reaction (10 µl) contained 5 µl of TaqMan™ Universal PCR Master Mix (Applied Biosystems), 2 µl of DNA (or sterile distilled water (SDW) for no template control (NTC)), 100 nM of the probe, and 300 nM of each forward and reverse primer. The PCR cycling conditions included 10 min of initial denaturation at 95 °C, followed by 40 cycles of denaturation for 30 s at 95 °C and annealing at 61 °C for 60 s. All qPCR assays were performed in duplicate, from which mean values were derived.

### Digital PCR assay optimization

Digital PCR assays were optimized for annealing temperature and primer and probe concentrations using the Quant Studio 3D digital PCR system (Applied Biosystems, USA) and the Absolute Q™ MAP16 Plate Kit (Cat No. A52865, Applied Biosystems). Initially, five annealing temperatures (58, 59, 60, 61, and 62 °C) were tested with a fixed primer/probe concentration of 300/100 nM. Thereafter, different primer concentrations (300, 600, and 900 nM) and probe concentrations (100, 150, 200, and 250 nM) were tested (12 combinations). Each reaction mixture was prepared in a total volume of 10 μl containing 2 μl of DNA Digital PCR Master Mix, 2 μl of genomic DNA (or SDW for NTC), and the required concentrations of probe and primers. Subsequently, 9 μl of this mixture was loaded into individual wells, and 15 μl of isolation buffer (Cat no: A52730, Applied Biosystems) was added to each reaction well. The wells were then sealed with gasket strips. The thermal cycling conditions were as follows: preheating at 96 °C for 10 min, 40 cycles of 96 °C for 15 s, and 60 °C for 1 min. Positive and negative partition data were acquired by applying a manual fluorescence threshold using Quanta Soft software V1.1, which provided the absolute copy count per microliter. All dPCR assays were performed in duplicate, from which mean values were derived.

### Linearity, specificity, and analytical sensitivity of qPCR and dPCR

To determine assay linearity and analytical sensitivity, experiments were conducted using serial DNA dilutions prepared from *F. langsethiae* (strain 201059), *F.* poae (strain 202172), and *F. sporotrichioides* (strain 89/N) (Table S1). Fivefold serially diluted genomic DNA ranging from 5000 to 1.6 pg/µl were used to generate the standard curve for each assay. For the *Tri5* assay, DNA dilutions of all three *Fusarium* species were tested, whereas for species-specific assays, DNA dilutions prepared from the corresponding target species were used. qPCR standard curves were generated by plotting log_10_ gDNA dilutions against the corresponding Cq values. The PCR efficiency of each assay was estimated from the slope of the corresponding standard curve using the following equation: *E* = −1 + 10^(−1/slope)^. For dPCR, log_10_ gDNA dilutions were plotted against copy number. To evaluate the analytical sensitivity of the qPCR and dPCR assays, serial dilutions of fungal DNA (0.35 to 24 pg) were prepared and used in the assays. Probit analysis was conducted to estimate the limit of detection (LoD) of the assays with a 95% probability [34]. The limit of quantification (LoQ) was determined as the minimum concentration at which replicates were positive with a 25% coefficient of variation (CV%) for the measured copies in dPCR [40].

### Effect of oat DNA on target estimation by qPCR and dPCR

To investigate the influence of oat DNA on assay estimates, samples containing a fixed amount of fungal DNA and various concentrations of oat grain DNA (5–50 ng) were prepared and tested using qPCR and dPCR. For both the *Tri5* and *Fl* assays, the fungal DNA used was 100 pg of *F. langsethiae* (strain 201059) DNA. Samples contained 40 pg of *F. poae* (strain 202172) DNA for the *Fp* assay and 200 pg of *F. sporotrichioides* (strain 89/N) DNA for the *Fs* assay. Fungal DNA mixed with nuclease-free water was used as a positive control, and SDW was used as NTC. In qPCR, Cq values were utilized to estimate the absolute concentration (pg) of the target by correlating them with the corresponding standard curves obtained from the fivefold dilutions of fungal DNA mentioned above. In contrast, the absolute copies of the target were directly determined by dPCR. For each sample, four replicates were tested in two consecutive experiments, and mean values were derived.

### dPCR and qPCR analysis of field samples

DNA extracts from the 20 field oat samples collected across Ireland were analyzed using dPCR and qPCR assays for *Tri5*, *F. langsethiae*, *F. sporotrichioides*, and *F. poae*. The quantification of targets using qPCR and dPCR was carried out under optimized conditions, with reactions containing 10 ng of field oat sample DNA (or SDW for NTC) and assays conducted as described above. Each sample was tested in two independent assays, each containing four technical replicates per sample and mean values were calculated.

For the *F. poae* assay, extrapolation from qPCR to dPCR (converting DNA concentrations from pg/µl to copies/µl) was performed specifically for samples that were positive in qPCR but negative in dPCR. This extrapolation provided an estimate of the number of target sequence copies in these samples, enabling a comparison of the sensitivity of the two methods. The conversion was performed using the equation described in [41], with the genome size of *Fusarium poae* taken as 39.3 Mb [42].

$$\frac{\text{DNA amount }(\text{g}) * 6.022 \times {10}^{23} (\text{copy}/\text{mol}) }{(\text{DNA length }(\text{bp}) * 660 (\text{g}/\text{mol}/\text{bp}) }$$

The formula calculates the total number of genomes in a given amount of DNA based on the genome size. To determine the number of target sequences, the total number of genome copies was multiplied by the rDNA copy number/per genome. Note, the rDNA copy number was found to range between 1 and 24 copies per genome, based on the analysis of eight sequenced strains of *F. poae* (GenBank IDs: GCA_001675295.1, GCA_030719125.1, GCA_013623615.1, GCA_019609905.1, GCA_030719135.1, GCA_030719155.1, GCA_030719145.1, and GCA_030719115.1), as identified via BLASTN analysis [43]. Due to this variability in rDNA copy number, the extrapolation from qPCR to dPCR was calculated across the range (1–24 copies), rather than relying on a single value.

Pearson’s correlation analysis was performed to determine the relationship between target estimates of dPCR (copies/µl) and qPCR assays (pg/µl). For both qPCR and dPCR, concentrations were calculated based on the target DNA detected in the reaction and normalized to the reaction volume. Cohen’s kappa coefficient (*κ*) was calculated to estimate the agreement between the two quantification techniques [44]. As detailed by [45], *κ* measures agreement between two methods as follows: below 0.00 is considered poor; 0.00 to 0.20 is slight; 0.21 to 0.40 is fair; 0.41 to 0.60 is moderate; 0.61 to 0.80 is substantial; and 0.81 to 1.00 is almost perfect.

### Statistical analysis

All statistical analyses were performed using SPSS v.27.0 (IBM Corp., Armonk, NY), whereas linear regression analysis for qPCR and dPCR was performed using Microsoft Excel 2021 (Microsoft, USA). The significance of differences between treatments was determined using one-way analysis of variance, and Tukey’s test was applied for post hoc analysis. To ensure transparency and reproducibility, this study followed the dMIQE and qMIQE guidelines [46, 47] for digital and quantitative PCR assays. Accordingly, we have reported assay design, validation parameters (sensitivity, specificity, efficiency, and dynamic range), reaction conditions, technical replicates, and controls (NTCs, positive controls). Additionally, data analysis, including normalization and result interpretation, has been presented following these guidelines.

## Results

### Adaptation of existing qPCR assays

**Fig. 1:**
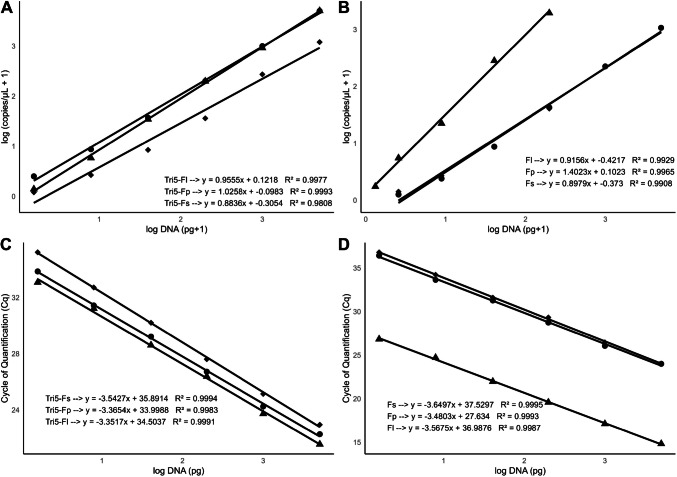
Standard curves relating fungal DNA concentrations to the corresponding results obtained via dPCR (copies/reaction) and qPCR (pg). Graphs represent qPCR and dPCR results, respectively, for **A**, **C** *Tri5* and **B**, **D** specific *Fusarium* species. DNA used for the *Tri5* assay was from *F. langsethiae* (*Tri5*-*Fl*), *F. poae* (*Tri5*-*Fp*), and *F. sporotrichioides* (*Tri5*-*Fs*), while the species DNA was used for the species-specific assays. See Table 2 for species used for each assay. The graphs show linear regression analysis and association equations. Shapes used for mean values represented: diamond, *Tri5-Fs* and *Fs*; triangle, *Tri5-Fp* and *Fp*; circle, *Tri5*-Fl and *Fl*

The previously published qPCR assays for trichothecene-producing *Fusarium* species and specific assays for *F. langsethiae*, *F. poae*, and *F. sporotrichioides* were initially evaluated for specificity and linearity using the Quant Studio 7 flex system (Applied Biosystems). The *Tri5* assay successfully detected all tested strains of *F. langsethiae*, *F. poae*, *F. graminearum*, *F sporotrichioides*, and *F. culmorum* (Table 1). The *Fl*, *Fp*, and *Fs* species-specific assays only amplified the target DNA of *F. langsethiae*, *F. poae*, and* F sporotrichioides,* respectively. All assays were negative for non-trichothecene-producing *M. nivale* and *F. avenaceum* strains. The linearity of the assays was determined using fivefold serial dilutions of *Fusarium* gDNA ranging from 5000 to 1.6 pg (conducted for one isolate each for species-specific assays and one per species for *Tri5*; Table 2)*.* Cq values obtained from qPCR assays were plotted against the input DNA concentrations, and regression analysis (Table 2; Fig. 1) indicated that all tested qPCR assays demonstrated good linearity with high coefficients of determination (*R*^*2*^ > 0.99). The primer efficiency was determined from the slope of the standard curves, resulting in PCR efficiencies of 94–98% for *Tri5*, 90.6% for *Fl*, 93.8% for *Fp*, and 88% for *Fs*.

**Table 1:** Specificity of qPCR and dPCR assays targeting the *Tri5* gene of *F. langsethiae* (*Fl*), *F. poae* (*Fp*), and *F. sporotrichioides* (*Fs*)

Fungal species/plant DNA	Fungal strain/oat cultivar	Signals in qPCR^1^				Signals in dPCR^1^			
		*Tri5*	*Fl*	*Fp*	*Fs*	*Tri5*	*Fl*	*Fp*	*Fs*
*Fusarium langsethiae*	201059 Fe 2390 Fe 2391	+ + +	+ + +	- - -	- - -	+ + +	+ + +	- - -	- - -
*F. poae*	202172 HUPO4 L1B	+ + +	- - -	+ + +	- - -	+ + +	- - -	+ + +	- - -
*F. sporotrichioides*	89/N DSM62425	+ +	- -	- -	+ +	+ +	- -	- -	+ +
*F. graminearum*	HUGR-6 L6 074 CC-19	+ + + +	- - - -	- - - -	- - - -	+ + + +	- - - -	- - - -	- - - -
*F. culmorum*	HUCU1 069 FC53	+ + +	- - -	- - -	- - -	+ + +	- - -	- - -	- - -
*F. avenaceum*	I103A I98C PO4	- - -	- - -	- - -	- - -	- - -	- - -	- - -	- - -
*M. nivale*	2746 44/3/M M7B	- - -	- - -	- - -	- - -	- - -	- - -	- - -	- - -
Oats DNA	Cultivar Husky	-	-	-	-	-	-	-	-

^1^“+” represents a positive fluorescence signal; “-” represents no fluorescence signal

**Table 2:** Linearity and analytical sensitivity of *Tri5* and species-specific qPCR and dPCR assays using fungal DNA extracts

Assay	Species and strains used^1^	qPCR^1^				dPCR^1^			
		*R*^2^	Slope	PE (%)	LOD	*R*^2^	Slope	LOD	LOQ
*Tri5*	*Fl* (201059)	0.99	−3.35	98.84	172	0.99	0.96	69	147
*Tri5*	*Fp* (202172)	0.99	−3.36	98.44	369	0.99	1.03	71	137
*Tri5*	*Fs* (89/N)	0.99	−3.54	91.64	443	0.99	0.88	75	188
*Fl-*specific	*Fl* (201059)	0.99	−3.57	90.6	800	0.99	0.92	71	383
*Fp*-specific	*Fp* (202172)	0.99	−3.48	93.8	5.8	0.99	1.4	5	27
*Fs*-specific	*Fs* (89/N)	0.99	−3.65	88	416	0.99	0.89	174	513

^1^Abbreviations: *Fl*, *F. langsethiae*; *Fp*, *F. poae*; *Fs*, *F. sporotrichioides*; *R*^2^, coefficient of determination; *PE (%)*, primer efficiency; *LOD*, limit of detection (fg/μl); *LOQ*, limit of quantification (fg/μl)

### Optimisation of the dPCR assays

The dPCR assays used the same primer/probe combinations as was used in qPCR. dPCR assays were optimized by determining the optimum annealing temperature and appropriate primer-probe concentrations to achieve better discrimination between partitions with high fluorescence (positives) containing the PCR amplification product and those exhibiting low fluorescence (negatives). This optimization also aimed to minimize the presence of “rain” partitions, which have intermediate fluorescence levels between positive and negative. Of the five annealing temperatures tested (58, 59, 60, 61, and 62 °C), the best separation of partitions for the *Tri5*, *Fl*, and *Fs* assays occurred at 60 °C, whereas 59 °C was found to be the optimum temperature for the *Fp* assay (Fig. S1). Thereafter, twelve different combinations of primer/probe concentrations were tested to select the optimal combinations for dPCR in terms of the separation of positive and negative partitions and reduction of the number of rain partitions. Based on the results (Fig. S1), the optimal primer probe concentrations (nM/nM) chosen for the *Tri5*, *Fl*, *Fp*, and *Fs* assays were 600/200, 300/200, 600/150, and 600/250, respectively.

dPCR demonstrated similar specificity to qPCR for all four assays (Table 1) and showed good linearity between input DNA concentrations and copies measured by dPCR for the *Tri5*, *Fl*, and *Fs* assays (*R*^*2*^ > 0.99; Table 2; Fig. 1). However, dPCR failed to accurately quantify high concentrations of *F. poae* DNA (1000 and 5000 pg) due to complete saturation with positive partitions (~20,000). Therefore, the linearity measurement between input DNA and dPCR copies was restricted to the DNA concentration range of 300 fg to 200 pg, resulting in an *R*^*2*^ value of 0.99.

### Comparison of the sensitivity of qPCR and dPCR using fungal DNA extracts

In terms of sensitivity, the limit of detection (LOD) at 95% probability was determined by assessing the signals obtained from serial dilutions of *Fusarium* gDNA using probit analysis. For all assays, LOD was tested using twofold serial dilutions ranging from 2000 to 63 fg/µl fungal DNA extract, while for the *Fp* assay, it was tested using fivefold serial dilutions ranging from 400 to 4.2 fg/µl (Table 2). In qPCR, the LOD values for the *Tri5* assay were 172, 369, and 443 fg/μl for *F. langsethiae*, *F. poae*, and *F. sporotrichioides*, respectively. For the species-specific assays (*Fl*, *Fp*, and *Fs*), the LOD values were 800, 5.8, and 416 fg/μl for *F. langsethiae*, *F. poae*, and* F sporotrichioides*, respectively. Comparatively, dPCR assays exhibited increased sensitivity with lower LOD values of 69, 71, and 75 fg/μl for *F. langsethiae*, *F. poae*, and *F. sporotrichioides*, respectively, in the *Tri5* assay. Similarly, specific dPCR assays for *Fl*, *Fp*, and *Fs* showed lower LOD values than qPCR (71, 5, and 174 fg/μl fungal DNA extract, respectively). This comparison demonstrated that dPCR generally exhibits higher sensitivity (lower LOD) than qPCR across most assays for pure fungal DNA extracts. The most significant difference was observed in the *Fl* assay, where dPCR was 11-fold more sensitive than qPCR, followed by the *Fs* assay, where dPCR was 2.5-fold more sensitive. For the *Tri5* assay, dPCR exhibited 2.5 to 6-fold higher sensitivity for detecting pure DNA from *F. langsethiae*, *F. poae*, and *F. sporotrichioides,* compared with qPCR. The LOQ values for the dPCR assays (Table 2) met the criteria for a LOQ (CV≤25%), indicating that the assays provide reliable and consistent results at the quantification limit. This ensured that the assays could measure concentrations with acceptable precision.

### The influence of plant DNA on target estimation by qPCR and dPCR

Since our goal was to develop assays that can be used to detect pathogens in oats, it was important that their sensitivity was not greatly impacted by the presence of plant DNA. Hence, we investigated the influence of oat genomic DNA on the quantification of assay targets by analyzing samples containing fungal DNA spiked with varying concentrations of oat grain DNA (Table 3). In the *Tri5* qPCR assay, the presence of plant DNA hindered qPCR estimation when the concentration exceeded 20 ng (*P < 0.05*). Conversely, in dPCR, an overestimation of *Tri5* copies occurred above 30 ng of plant DNA (*P* < 0.05). For the species-specific qPCR assays, a significant overestimation of targets was observed when the plant DNA concentration exceeded 10 and 40 ng for *Fl* and *Fp*, respectively (*P* < 0.05), while oat DNA had no noticeable effect on target estimation for *Fs*. In contrast, for dPCR, plant DNA had no significant influence on target estimation in any of the three species-specific assays (Table 3).

**Table 3:** Influence of oat grain DNA concentration on target estimation by qPCR and dPCR

Amount of oat grain DNA (ng)	DNA concentration measured by qPCR (pg/µl) (Mean ± SE)^1^				Copies measured by dPCR (copies/µl) (Mean ± SE)^1^			
	*Tri5* assay	*Fl* assay	*Fp* assay	*Fs* assay	*Tri5* *assay*	*Fl* assay	*Fp* assay	*Fs* assay
50	63 ± 1^c^	168 ± 3^b^	59 ± 6^c^	293 ± 43^a^	120 ± 1^c^	23 ± 2^a^	597 ± 31^a^	19 ± 1^ab^
40	72 ± 6^bc^	165 ± 9^b^	49 ± 6^ab^	331 ± 64^a^	118 ± 2^bc^	22 ± 1^a^	765 ± 44^a^	17 ± 1.5^ab^
30	81 ± 3^bc^	163 ± 5^b^	48 ± 2^ab^	268 ± 31^a^	114 ± 4^a^	23 ± 0.6^a^	670 ± 56^a^	19 ± 1.5^ab^
20	83 ± 2^abc^	151 ± 4^b^	49 ± 2^ab^	275 ± 56^a^	113 ± 2^ab^	23 ± 2^a^	774 ± 79^a^	21 ± 1^b^
10	92 ± 2^ab^	159 ± 6^b^	45 ± 2^a^	258 ± 21^a^	110 ± 1^ab^	21 ± 1^a^	703 ± 18^a^	17 ± 0.7^ab^
5	89 ± 9^ab^	115 ± 1^a^	36 ± 1^a^	266 ± 21^a^	103 ± 2^a^	25 ± 1^a^	614 ± 57^a^	17 ± 0.4^a^
Control	105 ± 9^a^	109 ± 3^a^	39 ± 0.4^a^	238 ± 17^a^	103 ± 1^ab^	24 ± 0.5^a^	804 ± 24^a^	16 ± 0.5^a^

^1^In each assay, values followed by different letters are significantly different according to the Tukey’s test (*P* < 0.05)

### Comparing qPCR and dPCR for the detection and quantitation of Fusaria in oat grain field samples

**Fig. 2:**
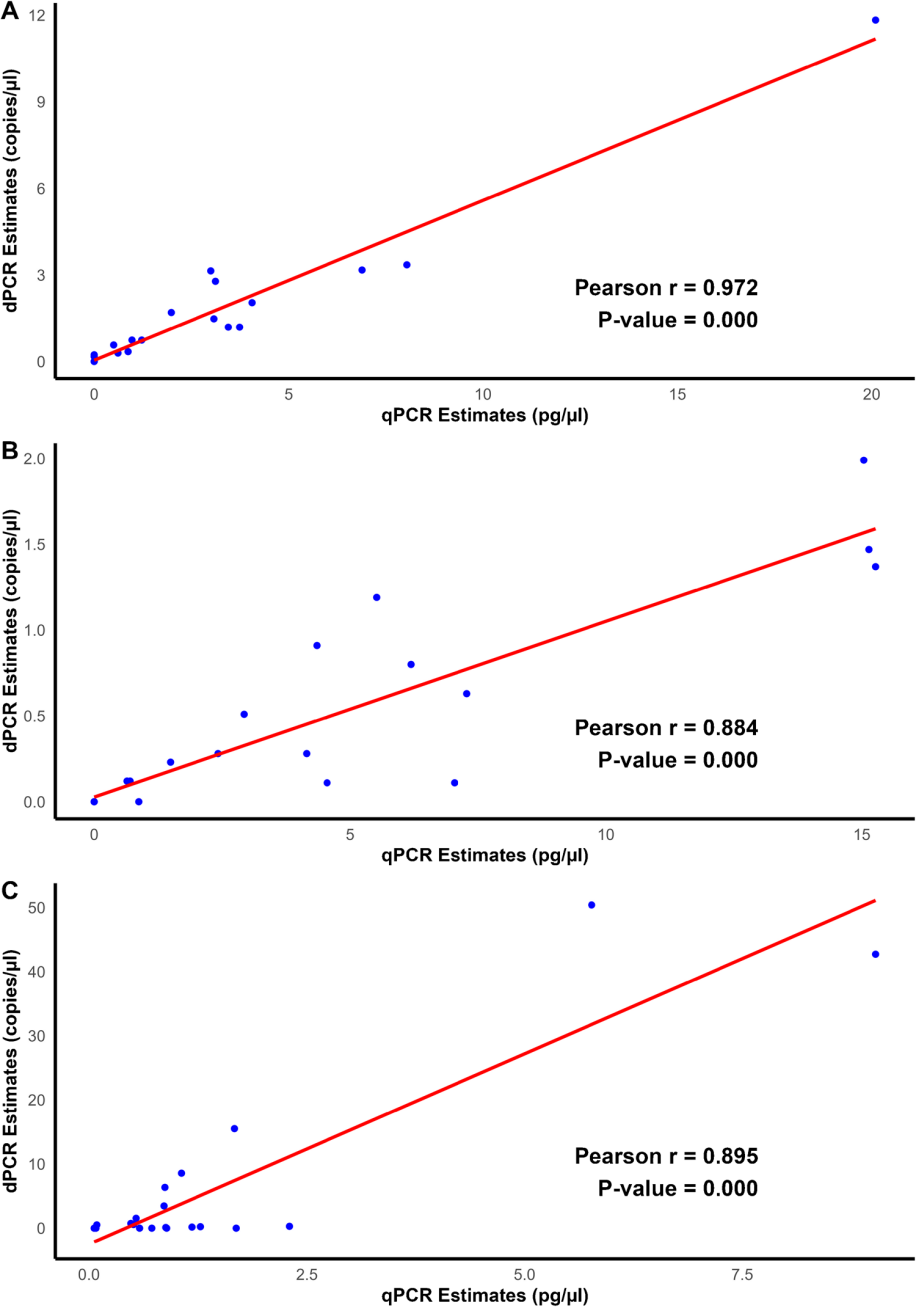
Pearson’s correlation plots comparing dPCR and qPCR estimates for 20 field-grown oat samples. Panel **A** *Tri5* assay, **B** *F. langsethiae* assay, and **C** *F. poae* assay. The red line in each panel represents the linear regression fit between the dPCR and qPCR estimates. The Pearson correlation coefficient (*r*) quantifies the strength of the linear relationship between the two methods, and the *p-*value indicates the statistical significance of the correlation

Tables 3 and 4 detail the concentration ranges and incidence of fungi detected in 20 oat field samples, as determined in each assay. Out of 20 field samples, 15 tested positive for *Fusarium* species using the *Tri5* assay in both qPCR and dPCR, with substantial agreement between the two methods, as indicated by a Cohen’s kappa (*κ*) value of 0.69. This *κ* value suggests that both qPCR and dPCR provide comparable and reliable results for *Fusarium* species detection using the *Tri5* assay. qPCR detected *Fusarium* DNA levels in positive samples ranging from 0.5 to 20.1 pg/µl, while dPCR detected target copies ranging from 0.17 to 11.83 copies/µl. Furthermore, the Pearson correlation coefficient between the estimates obtained using the two methods was strong (*r* = 0.97; *P < 0.001*), confirming the consistency of the results across both platforms (Fig. 2).

**Table 4:** Quantification of *Fusarium* species in oats samples (*n* = 20) collected from Irish field using qPCR and dPCR

Assay	qPCR			dPCR		
	Mean (pg/µl)	Range (pg/µl)	Positive samples (%)	Mean (copies/µl)	Range (copies/µl)	Positive samples (%)
Tri5 assay	3.8	0–20.1	15 (75)	1.75	0–11.83	17 (85)
*Fl* assay	4.68	0–15.27	16 (80)	0.5	0–2	15 (75)
*Fp* assay	1.53	0–9.03	20 (100)	6.56	0–50.4	14 (70)
*Fs* assay	-	-	0 (100)	0.05	0–0.23	6 (30)

For the *Fl* assay, 16 out of the 20 field samples tested positive for *F. langsethiae* using qPCR, with DNA levels ranging from 0.64 to 15.27 pg/µl (Tables 3 and 4). Similarly, dPCR detected 15 out of 20 samples as positive, with target copies ranging from 0.11 to 2 copies/µl. The agreement between qPCR and dPCR for this assay was nearly perfect (*κ* = 0.86), highlighting the strong reliability and consistency of the *Fl* assay results across both methods. Additionally, a strong positive correlation (*r* = 0.88, *P < 0.001*) was observed between the methods, indicating close agreement in the detection of *F. langsethiae*.

All 20 field samples tested positive for *F. poae* using the *Fp* qPCR assay, with DNA levels ranging from 0.06 to 9.03 pg/µl. In contrast, dPCR detected 14 out of 20 samples as positive, with target estimates ranging from 0.11 to 50.4 copies/µl (Tables 3 and 4). The six samples that tested negative by dPCR were at the low to moderate end of the concentration range detected by qPCR: 0.06 to 1.69 pg/µl. Most (5 of 8) sequenced *F. poae* genomes contain three copies of rDNA, and on this basis, the expected copy numbers for these samples in dPCR would be 126 per ul, which is above the LOD for dPCR (0.35/ul). Note it would also be well above the LOD for genomes with 1 or 24 copies of rDNA. Therefore, in terms of sensitivity, qPCR outperformed dPCR by detecting *F. poae* in all tested field samples. Cohen’s kappa coefficient analysis confirmed the agreement between the two methods was poor (*κ* = 0.00). However, the correlation between the estimates from both methods remained relatively strong (*r* = 0.89, *P* < 0.001), suggesting a linear relationship despite discrepancies in the sensitivity of *Fp* detection within field samples.

The *Fs* qPCR assay did not detect the pathogen in any of the 20 field samples, while dPCR identified 6 out of 20 samples as positive for *F. sporotrichioides*, ranging from 0.11 to 0.23 copies/µl (Tables 3 and 4). These values are below the LOD for the *Fs* dPCR assay (174 fg/µl, corresponding to 4.23 genome copies/µl); thus, dPCR is detecting extremely low concentrations of *Fs* DNA, below the LOD. The agreement between the two methods was poor (*κ* = 0.00), suggesting that dPCR has higher sensitivity for detecting *F. sporotrichioides* in field samples than qPCR (Table 5).

**Table 5:** Detection capability of *Fusaria in* field samples using dPCR and qPCR

Assay	Number of samples (out of a total of 20)				
	Positive by qPCR and dPCR	Negative by qPCR and dPCR	Positive by qPCR but not by dPCR	Positive by dPCR but not by qPCR	Cohen’s Kappa coefficient (*κ*)
*Tri5* assay	15	3	0	2	0.69
*Fl* assay	15	4	1	0	0.86
*Fp* assay	14	0	6	0	0.0
*Fs* assay	0	14	0	6	0.0

## Discussion

As stated in the introduction, dPCR has several advantages over traditional qPCR. Hence, these assays were developed to detect *Fusarium* infection of oats, which is not always reflected in visual disease symptoms. Building on previously published TaqMan®-based qPCR assays [28–30], we developed dPCR assays for the detection of all trichothecene-producing *Fusaria* and specific assays for the detection of the *Fusarium* species that commonly attack oats: *F. langsethiae*, *F. poae*, and *F. sporotrichioides*. We also demonstrated the suitability of dPCR for the detection of *Fusaria* in field oat samples. We optimized and compared both the qPCR and dPCR assays in our laboratory. Both quantification methods exhibited good linearity across a range of DNA dilutions. However, we observed saturation of positive partitions in dPCR for the *Fp*-specific assay, while qPCR maintained a linear dynamic range. This saturation in dPCR can be attributed to the presence of multiple copies of the target (rDNA) in the *Fusarium* genome. Similarly, an earlier study [48] reported that an excess concentration of the target resulted in positive saturation of the partitions, making the Poisson algorithm ineffective and leading to a reduced dynamic range with dPCR.

PCR assays targeting multi-copy sequences, including rDNA, are widely used for fungal detection due to their high sensitivity [49–52]. Species-specific PCR assays targeting the IGS region of rDNA have been developed to enhance sensitivity for detecting *F. poae*, *F. graminearum*, *F. culmorum*, *F. sporotrichioides*, and *F. equiseti* [53]. Similarly, a *F. poae*-specific TaqMan qPCR assay targeting rDNA has been applied to quantify fungal DNA in wheat grain, demonstrating high sensitivity and correlation with mycotoxin presence [54]. However, the variability in the number of rDNA repeats among strains presents challenges for precise absolute quantification [55]. This variability likely contributed to the observed saturation in dPCR results for *F. poae*. While this assay is highly sensitive and suitable for detection, it is less reliable for precise quantification due to the inherent variability in rDNA copy numbers. This variability can affect absolute quantification, particularly in dPCR, where the Poisson algorithm assumes a stable target copy number [56]. Despite this limitation, the assay remains a valuable tool for detecting *F. poae* in grain samples due to its high sensitivity and tolerance to PCR inhibitors. Dilution of high DNA concentration samples could effectively mitigate saturation effects, and importantly, no saturation was observed in field samples, confirming the assay’s practical applicability for detecting *F. poae* in real-world conditions.

PCR amplification can be challenging due to the presence of inhibitors in the samples, which can reduce PCR efficiency. The sample matrix and extraction procedures can introduce these inhibitors [57, 58]. Diluting the sample DNA concentration can help minimize these effects, provided the target DNA remains detectable [59, 60]. For example, a study [61] found that a 10-fold dilution was sufficient to remove inhibitors for *Phytophthora nicotianae* detection in dPCR, while qPCR required a 20-fold dilution. To better understand how oat DNA affects *Fusarium* quantification, we tested the impact of varying oat DNA concentrations on both qPCR and dPCR assays. Increasing the amount of oat DNA caused the overestimation of targets in the *Fl* and *Fp* qPCR assays, but this effect was not observed in dPCR. In the *Tri5* assay, oat DNA over 30 ng led to overestimation of the dPCR results, while qPCR underestimated the targets when oat DNA exceeded 20 ng. The *Fs* assay was unaffected by oat DNA in either method. Our study demonstrated that dPCR is less susceptible to variations in oat DNA concentration for *Fusarium* diagnostics. Similarly, a previous study [34] reported that dPCR assays were more tolerant than qPCR to various concentrations of extracts obtained from stem and root tissues for the detection of *Verticillium* wilt pathogens in alfalfa.

dPCR assays are often more sensitive than qPCR for diagnosing plant pathogens [48, 62–64]. For instance, dPCR offered improved analytical sensitivity over qPCR, enhancing the detection of *Ralstonia solanacearum* in potato tuber samples, even at lower concentrations [58]. Similarly, a dPCR assay for *Tilletia caries* with a detection limit 10 times lower than that of qPCR [65]. In our study, dPCR exhibited increased detection capability compared to qPCR for all assays tested except *Fp*, when considering both pure fungal DNA extracts and field oat samples. Cohen’s kappa coefficient (*κ*) analysis confirmed that, in contrast to *Fl* and *Tri5*, the *Fp* and *Fs* assays showed significant discrepancies in their detection capabilities. These variations can be attributed to the differing sensitivities of dPCR and qPCR, with dPCR more effective at detecting low levels of *F. sporotrichioides*, while qPCR was better at detecting *F. poae* at low to moderate levels in field samples. While dPCR is thus more sensitive than qPCR for detecting *Fs*, any quantitative results for samples near or below the LOD should be interpreted with caution, as results at these levels may be influenced by stochastic effects (random variation in partition distribution) and matrix-related issues in the field samples [66, 67]. Similarly, a previous study [63] reported that dPCR had a lower detection limit for the 16S rRNA gene of *Candidatus Liberibacter asiaticus* compared to qPCR, allowing for the detection of smaller quantities of the target gene.

qPCR and dPCR have demonstrated positive correlations in target estimates of other plant pathogens in field samples, as reported in studies by [68] and [39]. Despite the observed performance discrepancies in our study, strong positive correlations were observed between qPCR and dPCR for *Tri5*, *Fl*, and *Fp* field sample results. Similarly, a study [48] identified discrepancies in the detection capabilities of *Xanthomonas citri* subsp. *citri* (*Xcc*) in citrus samples using qPCR and dPCR; there was still a strong positive correlation between the two methods. In another study, dPCR exhibited higher sensitivity than qPCR for detecting *Verticillium dahliae* and *V. longisporum* in cotton root and soil samples; there were good correlations between the results for both methods [69].

In conclusion, this study successfully developed and optimized digital PCR (dPCR) assays for detecting and quantifying trichothecene-producing *Fusarium* species, including *F. langsethiae*, *F. poae*, and *F. sporotrichioides*, in oat grains. Both qPCR and dPCR were effective in detecting trichothecene-producing *Fusarium* species from pure DNA extracts and field samples. However, dPCR exhibited superior sensitivity for detecting *Tri5*, *Fl*, and *Fs* targets compared to qPCR. Notably, dPCR outperformed qPCR in identifying *F. sporotrichioides*, which was undetected by qPCR in field samples. For quantification, the choice of the target sequence most likely influenced accuracy. dPCR was precise for single-copy targets such as *TEF1α*, whereas quantification of *F. poae* using the rDNA assay could be affected by the multi-copy nature of the rDNA target, leading to saturation effects at high DNA concentrations. In future work, selecting a single-copy target may improve the reliability of *F. poae* quantification in both dPCR and qPCR assays. dPCR offers key advantages, including greater resistance to DNA inhibitors and the ability to perform absolute quantification without requiring standard curves, making it a valuable diagnostic tool for Fusarium head blight (FHB) detection in oats. These assays are now being deployed as tools to study the control of FHB in oats and the epidemiology of this disease.

## Supplementary Information

Below is the link to the electronic supplementary material.

Supplementary file1 (DOCX 302 KB)

Supplementary file2 (DOCX 30 KB)

## Data Availability

All data generated from this study are included in this article and its supplementary files.
