# Supplementary material for: Digital PCR assays for quantifying trichothecene-producing Fusarium species, including Fusarium langsethiae, F. poae, and F. sporotrichioides, in oats
Source: Anal Bioanal Chem. 2025 Mar 21;417(13):2957–69. doi: 10.1007/s00216-025-05840-0 (PMC12052851; doi:10.1007/s00216-025-05840-0)
Supplement: Supplementary file 1 — Supplementary file1 (DOCX 302 KB) [file 216_2025_5840_MOESM1_ESM.docx]

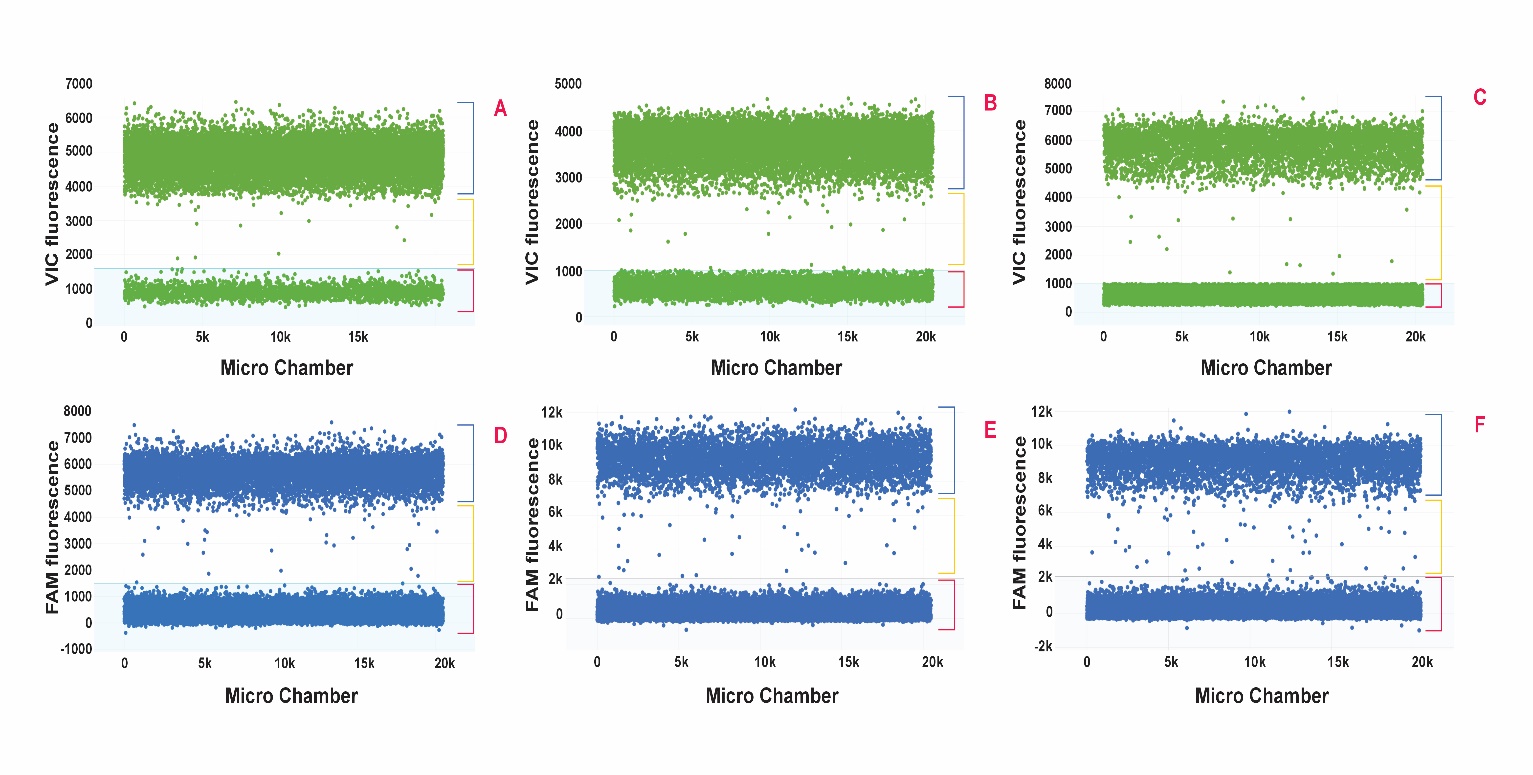


**Fig S1.** Representative digital PCR amplification plots for the *Tri5* assay (A, B, and C) and species-specific assay (D, E, and F). The *Tri5* assay utilized genomic DNA extracted from pure cultures of *F. langsethiae* (A), *F. poae* (B), and *F. sporotrichioides* (C), analysed at an annealing temperature of 60°C with a primer/probe combination of 600/200 nm. Species-specific assays (*Fl, Fp,* and *Fs*) employed genomic DNA extracts of specific targets, each analysed at the appropriate annealing temperature and primer/probe combination. (E) *Fl* assay amplification plot for *F. langsethiae* DNA (60°C and 300/200 nm). (F) *Fp* assay amplification plot for *F. poae* DNA (59°C and 600/150 nm). (G) *Fs* assay amplification plot for *F. sporotrichioides* DNA (59°C and 600/250 nm). Blue brackets represent positive droplets, red indicates negative droplets, and yellow represents intermediate droplets (rains).
