# Supplementary material for: Digital PCR assays for quantifying trichothecene-producing Fusarium species, including Fusarium langsethiae, F. poae, and F. sporotrichioides, in oats
Source: Anal Bioanal Chem. 2025 Mar 21;417(13):2957–69. doi: 10.1007/s00216-025-05840-0 (PMC12052851; doi:10.1007/s00216-025-05840-0)
Supplement: Supplementary file 2 — Supplementary file2 (DOCX 30 KB) [file 216_2025_5840_MOESM2_ESM.docx]

**Table S1.** Fungal strains used in this study.

| **Species** | **Strains** | **Country of origin** | **Source** |
| --- | --- | --- | --- |
| *Fusarium langsethiae* | Fl201059  Fe 2390  Fe 2391 | UK  UK  UK | NIBIO, Norway  FERA, UK  FERA, UK |
| *F. poae* | 202172  HUPO4  L1B | Norway  Hungary  Ireland | NIBIO, Norway  ABC, Hungary  SBES, UCD |
| *F. Sporotrichioides* | 89/N  DSM 62425 | Ireland  Germany | SBES, UCD  DSMZ, Germany |
| *F. graminearum* | HUGR-6  L6  074  CC-19 | Hungary  Ireland  Italy  Ireland | ABC, Hungary  SBES, UCD  Prof. Antonio Moretti, INRC  SBES, UCD |
| *F . avenaceum* | I103A  I98C  067 | Ireland  Ireland  Italy | SBES, UCD  SBES, UCD  Prof. Antonio Moretti, INRC |
| *F. culmorum* | HUCU3  069  FC53 | Hungary  Italy  UK | ABC, Hungary  Prof. Antonio Moretti, INRC  CSL, UK |
| *M. nivale* | 2744  44/3/M  M7B | Italy  UK  Ireland | Prof. Antonio Moretti, INRC  Prof. Paul Nicholson, JIC, UK  SBES, UCD |

Strains collected from our laboratory are available upon request, subject to institutional agreements. Strains obtained via MTAs from other institutes are not available for distribution; researchers should contact the respective institutes for access

**Table S2.** Primers and probes used in this study.

| **Primer/probe** | **Nucleotide sequences (5′-3′)** | **Target species** | **Target** | **Reference** |
| --- | --- | --- | --- | --- |
| TmfTriF^1^ | CAGCAGMTRCTCAAGGTAGACCC | Trichothecene-producing *Fusarium* species | Trichodiene synthase (*Tri5*) | Yli-Mattila et al. (2008) |
| TMFTriR^1^ | AACTGTAYACRACCATGCCAAC |  |  |  |
| TMTrip | VIC- AGCGACTACAGGCTTCCCTCCAAACAAT-MGB |  |  |  |
| EF1 FLF3 | GCCGTGTCGTAATTTTTTTTGTG | *F. langsethiae* | Translation elongation factor 1-α (EF1α) | Sonia *et al .* (2018) |
| EF1 FLR3 | AAATGGCTATGTGGGAAGGAAG |  |  |  |
| EF1_FL | FAM-GGGCTCATACCCCGCCACTCGA-MGB |  |  |  |
| TmPoae F | GCTGAGGGTAAGCCGTCCTT | *F. poae* | rDNA | Yli-Mattila et al. (2008) |
| TmPoae R | TCTGTCCCCCCTACCAAGCT |  |  |  |
| TMpoae probe | FAM-ATTTCCCCAACTTCGACTCTCCGAGGA-MGB |  |  |  |
| EF1-FS_F3 | GCAAGTCGACCACTGTGAGTAC | *F. sporotrichioides* | Translation elongation factor 1-α (EF1α) | Boutigny *et al .* (2019) |
| EF1-FS_R2 | GCGCCCATGTAAATGGATG |  |  |  |
| EF1 FS | FAM-TGGGAAGGGCAAAAGCGCCTGT-BHQ |  |  |  |

^1^Degenerate bases follow the IUPAC nucleotide code: M = A/C, R = A/G, Y = C/T, allowing the primer to accommodate sequence variability in trichothecene-producing *Fusarium* species

**Table S3.** Quantification of *Fusarium* species in oats samples (n=20) collected from Irish field using qPCR and dPCR

| **Assay** | **qPCR** | | | **dPCR** | | |
| --- | --- | --- | --- | --- | --- | --- |
|  | **Mean**  **(pg/µl)** | **Range**  **(pg/µl)** | **Positive samples (%)** | **Mean**  **(copies/µl)** | **Range**  **(copies/µl)** | **Positive samples (%)** |
| **Tri5 assay** | 3.8 | 0-20.1 | 15(75) | 1.75 | 0-11.83 | 17(85) |
| ***Fl* assay** | 4.68 | 0-15.27 | 16(80) | 0.5 | 0-2 | 15 (75) |
| ***Fp* assay** | 1.53 | 0-9.03 | 20(100) | 6.56 | 0-50.4 | 14 (70) |
| ***Fs* assay** | - | - | 0(100) | 0.05 | 0-0.23 | 6(30) |

**Table S4.** Detection capability of *Fusaria in* field samples using dPCR and qPCR

| **Assay** | **Number of samples (out of a total of 20)** | | | | |
| --- | --- | --- | --- | --- | --- |
|  | **Positive by qPCR and dPCR** | **Negative by qPCR and dPCR** | **Positive by qPCR but not by dPCR** | **Positive by dPCR but not by qPCR** | **Cohen's Kappa coefficient**  (*κ*) |
| ***Tri5* assay** | 15 | 3 | 0 | 2 | 0.69 |
| ***Fl* assay** | 15 | 4 | 1 | 0 | 0.86 |
| ***Fp* assay** | 14 | 0 | 6 | 0 | 0.0 |
| ***Fs* assay** | 0 | 14 | 0 | 6 | 0.0 |

**Table S5.** BLASTn analysis of the TEF1α gene across available genome assemblies in the NCBI database.

| Species | Strain ID | Genome Assembly Availability | TEF1α Copy Number | Query Coverage (%) | Identity (%) |
| --- | --- | --- | --- | --- | --- |
| *F. langsethiae* | Fe2391 | NCBI | Single-copy | 100 | 99.5 |
| *F. langsethiae* | MFG 217701 | NCBI | Single-copy | 100 | 99.6 |
| *F. langsethiae* | MFG 217702 | NCBI | Single-copy | 100 | 100 |
| *F. langsethiae* | Fl201059 | NCBI | Single-copy | 100 | 99.6 |
| *F. sporotrichioides* | S17/16 | NCBI | Single-copy | 100 | 99.5 |
| *F. sporotrichioides* | S18/43 | NCBI | Single-copy | 100 | 99.5 |
| *F. sporotrichioides* | S17/1 | NCBI | Single-copy | 100 | 99.5 |
| *F. sporotrichioides* | FB2 | NCBI | Single-copy | 100 | 99.8 |
| *F. sporotrichioides* | NRRL 3299 | NCBI | Single-copy | 100 | 99.7 |
